# Transumbilical Laparoscopic-Assisted Appendectomy (TULAA): A Fast, Safe, and Scarless Alternative to Conventional Three-Port Laparoscopic Appendectomy in Pediatric Patients

**DOI:** 10.7759/cureus.80505

**Published:** 2025-03-13

**Authors:** Satvik S Phutane, Vikram Sindagikar, Mallikarjun B Patil, Dayanand Biradar, Anand Suntan, Avinash Hanchinal

**Affiliations:** 1 General Surgery, Shri B. M. Patil Medical College Hospital and Research Centre, BLDE (Deemed to be University), Vijayapura, IND; 2 Surgery, Shri B. M. Patil Medical College Hospital and Research Centre, BLDE (Deemed to be University), Vijayapura, IND

**Keywords:** appendicitis, laparoscopic surgery, minimally invasive surgery, pediatric surgery, tulaa

## Abstract

Introduction: Acute appendicitis is a common pediatric surgical emergency requiring timely intervention. Conventional three-port laparoscopic appendectomy (CLA) is widely used, offering reduced postoperative pain, faster recovery, and lower wound infection rates. The transumbilical laparoscopic-assisted appendectomy (TULAA) technique, a single-incision approach, promises improved cosmetic outcomes, shorter operative time, and reduced morbidity. This study aims to compare TULAA and CLA in terms of surgical efficiency, postoperative pain, complications, hospital stay, conversion rate, and cosmetic acceptance.

Materials and methods: This comparative study was conducted at Shri B.M. Patil Medical College, Vijayapura, India, from September 2023 to September 2024. A total of 36 patients under 18 years with appendicitis were enrolled (18 in the TULAA group and 18 in the CLA group). Primary outcomes assessed included operative time, pain (using visual analogue scale, or VAS), complications, wound issues, hospital stay, conversion rate, and cosmetic acceptance.

Results: TULAA demonstrated a significantly shorter operative time (p<0.001) and lower postoperative pain (p=0.0009) compared to CLA. Hospital stay was shorter in the TULAA group (p=0.0008). While no significant difference in postoperative complications (p=0.1456) was observed, cosmetic acceptance was notably better in TULAA (p<0.001).

Conclusion: TULAA is a quick, safe, and cosmetically superior alternative to CLA in pediatric appendicitis, offering shorter operative time, reduced pain, and enhanced recovery.

## Introduction

Acute appendicitis remains one of the most common surgical emergencies worldwide, necessitating timely intervention to prevent complications [1]. The estimated lifetime risk of developing acute appendicitis ranges between 6.7% and 8.6% [2]. The evolution of minimally invasive surgical techniques has significantly influenced appendectomy procedures, with conventional three-port laparoscopic appendectomy (CLA) becoming the standard approach due to its well-documented benefits, including reduced pain postoperatively, fast recovery, and reduced wound infection rates [3]. However, recent advancements have introduced transumbilical laparoscopic-assisted appendectomy (TULAA), a single-incision technique that seeks to enhance patient outcomes by reducing the number of incisions while maintaining the advantages of laparoscopy​ [4].

TULAA employs a transumbilical approach, allowing extracorporeal mobilization and removal of the appendix while leveraging laparoscopic visualization for precision [5]. Proponents of this technique highlight its superior cosmetic results, shorter operative time, and potentially lower postoperative morbidity compared to CLA. Several systematic reviews and meta-analyses have demonstrated that TULAA offers comparable or even superior outcomes in terms of surgical efficiency, hospital stay, and intra-abdominal infection rates, making it a promising alternative for pediatric populations​ [6].

Despite its advantages, the widespread adoption of TULAA remains limited due to concerns regarding technical feasibility, learning curve, and potential complications such as incomplete mobilization of the appendix, necessitating conversion to a multi-port laparoscopic or open approach​ [7]. Comparative studies have reported mixed outcomes, with some indicating a higher rate of additional port placement in TULAA, while others suggest no significant difference in major postoperative complications between the two techniques​ [8].

We hypothesize that TULAA offers superior outcomes compared to CLA in terms of operative time, pain, and cosmetic acceptance while maintaining comparable complication rates. By synthesizing data from multiple studies, this research seeks to provide evidence-based recommendations for optimal surgical management of acute appendicitis, particularly in pediatric populations.

## Materials and methods

This comparative study was conducted in the Department of Surgery at Shri B. M. Patil Medical College Hospital and Research Centre, BLDE (Deemed to be University), Vijayapura, from September 2023 to September 2024. Patients under the age of 18 years presenting with appendicitis to the General Surgery Outpatient Department or Emergency/Casualty were included in the study. Exclusion criteria comprised patients diagnosed with appendicular phlegmon, appendicular abscess, or appendicular mass, as well as those who underwent conversion from laparoscopic to open appendectomy. Ethical clearance was obtained (IEC/913/2023-24), and informed consent was taken from all participants or their guardians before enrollment in the study.

**Figure 1:**
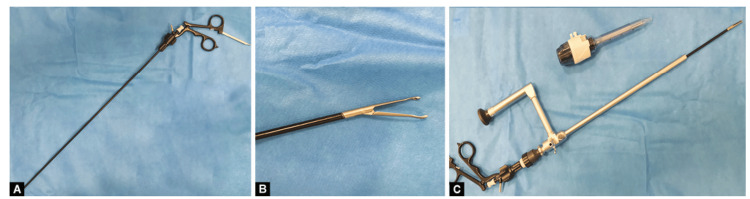
(A) The grasper, (B) the tip of the grasper, (C) the grasper inside the telescope with a 10mm port.

The study aimed to compare two surgical techniques for appendectomy: TULAA and CLA. Patients were allocated into two groups randomly based on the surgical procedure performed. Group 1 underwent single-port TULAA, while Group 2 underwent CLA. A structured proforma was used to record demographic details, clinical history, intraoperative findings, and postoperative outcomes (Figure 1).

**Figure 2:**
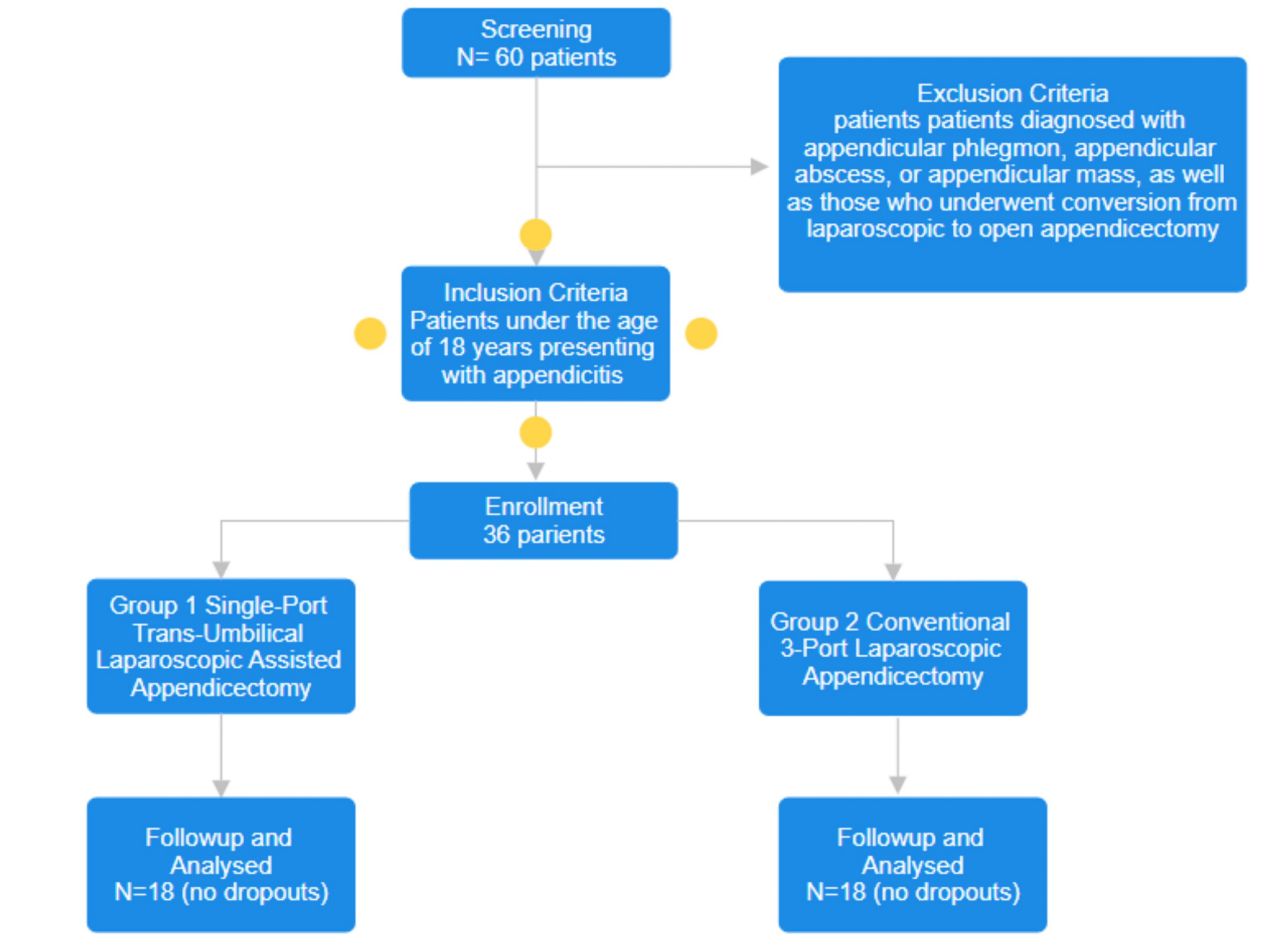
Consolidated Standards of Reporting Trials (CONSORT) flow diagram.

The primary parameters evaluated included operative time (from incision time to completion of the procedure), postoperative pain which was assessed using visual analogue scale (VAS), complications such as bleeding and infection, wound-related complications, hospital stay duration, that is, from time of surgery to discharge, conversion rate, and cosmetic acceptance which was assessed post-operatively as below average, average, good and excellent. The sample size was determined based on an anticipated mean and standard deviation of operative time in the two groups, requiring a minimum of 18 participants per group (total 36) to detect a true difference in means with 90% power and a 5% significance level. Effect sizes were considered to ensure the study was adequately powered to detect meaningful differences while minimizing type I and type II errors (Figure 2).

Data collected were entered into Microsoft Excel (Microsoft® Corp., Redmond, WA, USA) and analysed using Statistical Package for the Social Sciences (IBM SPSS Statistics for Windows, IBM Corp., Version 26.0, Armonk, NY). Descriptive statistics were presented as counts and percentages for categorical variables. The chi-square test was used for categorical variable comparisons between groups. A p-value of <0.05 was considered statistically significant.

## Results

The study included 18 cases and 18 controls, with the age distribution showing a higher proportion of younger participants (6-10 years) in the case group (100%) compared to the control group (11.11%). The majority of participants in both groups were aged 11-14 years (56% in cases, 44% in controls) and 15-18 years (29% in cases, 44% in controls), but the difference was not statistically significant (p = 0.329). Regarding sex distribution, females comprised 64% of the cases and 36% of the controls, while males constituted 41% of the cases and 59% of the controls (p = 0.1714) (Table 1).

**Table 1:** General characteristics. The data has been represented as N(%). A p-value of <0.05 was considered statistically significant.

Characteristics		Cases (N = 18)	Control (N = 18)	Statistical value
Age group	6 to 10 years	4 (100%)	2 (11.11%)	Chi-square value = 2.22; p-value = 0.329
	11 to 14 years	10 (56%)	8 (44%)	
	15 to 18 years	4 (29%)	8 (44%)	
Sex	Female	9 (64%)	5 (36%)	Chi-square value = 1.87; p-value = 0.1714
	Male	9 (41%)	13 (59%)	

**Figure 3:**
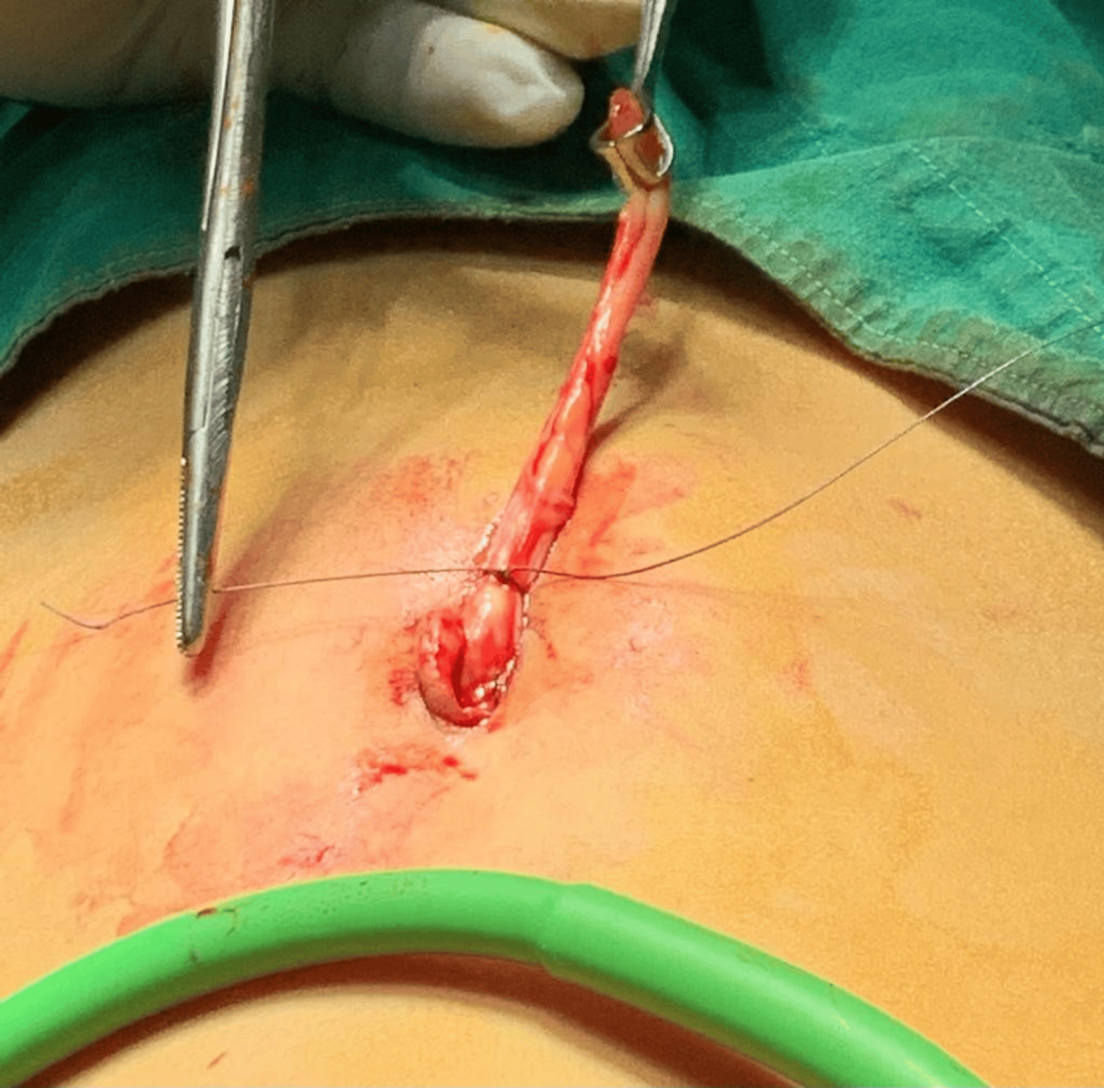
The base of the appendix being securely transfixed using a synthetic absorbable monofilament braided polyglactin 2-0 round body suture.

The diagnosis distribution was comparable between groups (p = 0.9195). Operative time was significantly shorter in the case group (p < 0.001), with all cases completing surgery within 30 minutes, whereas all controls required more than 30 minutes (Table 2 and Figure 3).

**Table 2:** Diagnosis and operative time. The data has been represented as N(%). A p-value of <0.05 was considered statistically significant.

Characteristics		Cases (N = 18)	Control (N = 18)	Statistical value
Diagnosis	Acute appendicitis	6 (46%)	7 (54%)	Chi-square value = 0.16; p-value = 0.9195
	Recurrent appendicitis	6 (55%)	5 (45%)	
	Subacute appendicitis	6 (50%)	6 (50%)	
Operative time	≤15 mins	3 (100%)	0 (0%)	Chi-square value = 32.25; p-value ≤ 0.001
	15-30 mins	15 (94%)	1 (6.3%)	
	30-45 mins	0 (0%)	6 (100%)	
	>45 mins	0 (0%)	11 (100%)	

Postoperative pain was significantly lower in cases (p = 0.0009), with 92% reporting a pain score of 2 compared to 8.3% in controls, while all cases with a pain score of 6 belonged to the control group. Hospital stay was significantly less in cases (p = 0.0008), with 76% discharged within three days compared to 24% in controls. Postoperative complications were noted in two cases (p = 0.1456) (Table 3).

**Table 3:** Postoperative outcomes. The data has been represented as N(%). A p-value of <0.05 was considered statistically significant.

Postoperative outcomes		Cases (N = 18)	Control (N = 18)	Statistical value
Postoperative pain	Score 2	11 (92%)	1 (8.3%)	Chi-square value = 16.47; p-value = 0.0009
	Score 3	4 (57%)	3 (43%)	
	Score 4	3 (25%)	9 (75%)	
	Score 6	0 (0%)	5 (100%)	
Postoperative complications		2 (100%)	0 (0%)	Chi-square value = 2.11; p-value = 0.1456
Duration of hospital stay	<3 days	16 (76%)	5 (24%)	Chi-square value = 14.13; p-value = 0.0008
	3-5 days	1 (9.1%)	10 (91%)	
	>5 days	1 (25%)	3 (75%)	

**Figure 4:**
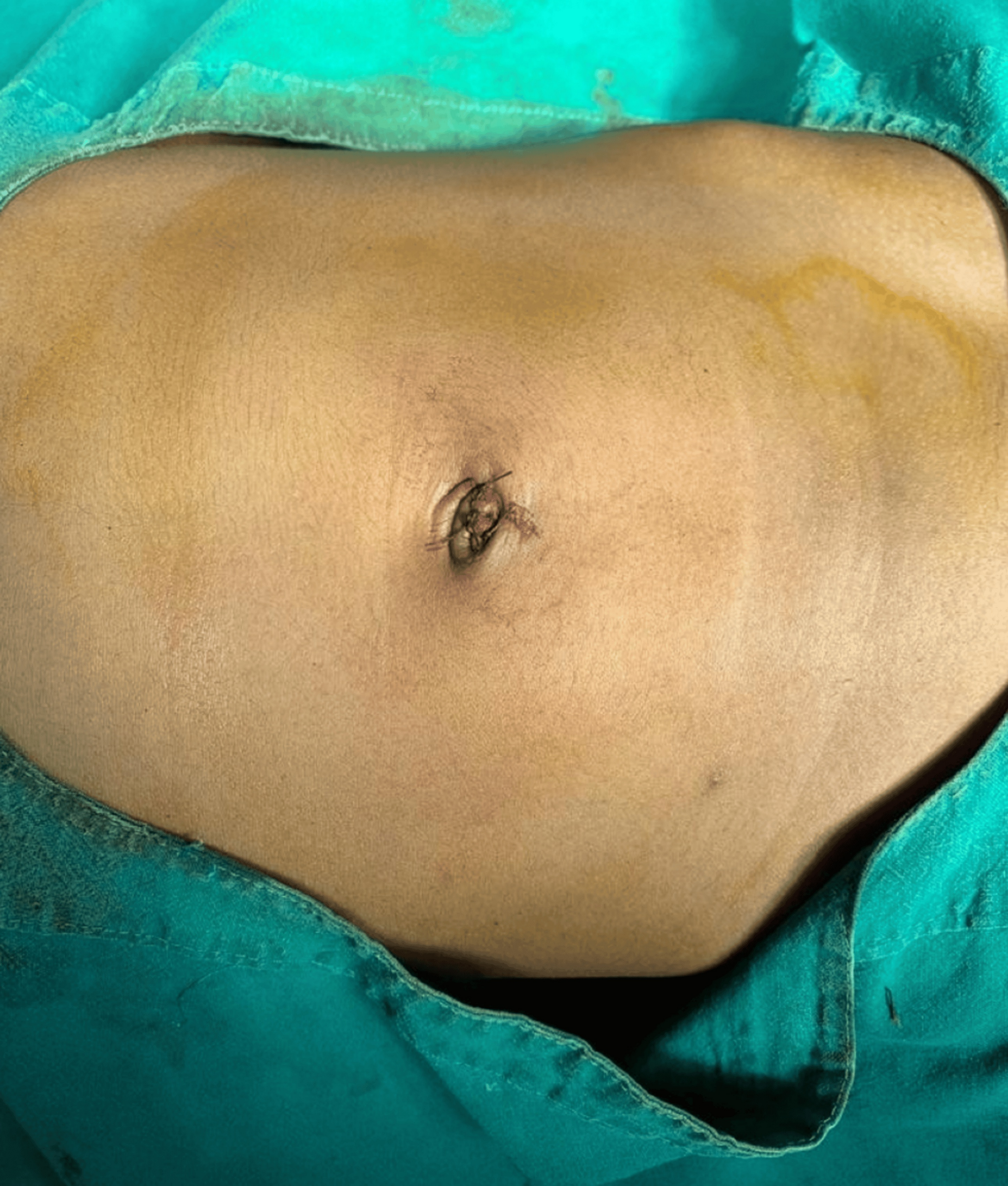
Immediate postoperative image.

Cosmetic acceptance was significantly better in cases (p < 0.001), with all cases rating their outcome as good or excellent, while all controls rated it as average. Conversion was required in two controls but none in cases (p = 0.1456) (Table 4 and Figure 4).

**Table 4:** Cosmetic acceptance and conversion rate. The data has been represented as N(%). A p-value of <0.05 was considered statistically significant.

Cosmetic acceptance and conversion rate		Cases (N = 18)	Control (N = 18)	Statistical value
Cosmetic acceptance	Average	0 (0%)	18 (100%)	Chi-square value = 36; p-value ≤ 0.001
	Good	13 (100%)	0 (0%)	
	Excellent	5 (100%)	0 (0%)	
Conversion	Converted	0 (0%)	2 (100%)	Chi-square value = 2.12; p-value = 0.1456
	No	18 (53%)	16 (47%)	

## Discussion

The present study consisted of 18 cases and 18 controls, with a higher proportion of younger participants (6-10 years) in the case group (100%) compared to the control group (11.11%). In contrast, Bindi et al. (2023) reported a mean age distribution across different pediatric age groups, with a total sample of 181 patients [9], whereas Go et al. (2016) examined patients aged under 15​​ [7]. Similarly, Noviello et al. (2015) reported a mean age of 9.2 years, which aligns more closely with the current study​ [10]. Regarding sex distribution, females constituted 64% of cases in the present study, while males accounted for 41% of cases, though the difference was not statistically significant (p = 0.1714). Similar trends have been reported in pediatric appendectomy studies, with Bindi et al. (2023) [9] and Noviello et al. (2015) [10] showing a slight male predominance in their cohorts​.

One of the significant findings of the present study is that the operative time was significantly less in the cases (p < 0.001), with all cases completing surgery within 30 minutes. This is in stark contrast to previous studies. Bindi et al. (2023) found that TULAA had a significantly shorter operative time than CLA, with mean times of 56.4 minutes versus 70.9 minutes, respectively (p < 0.0001)​ [9]. Similarly, Cheema et al. (2024) reported that TULAA reduced operative time compared to CLA by approximately 11.16 minutes (p < 0.00001)​ [11]. On the other hand, Wu et al. (2022) reported that single-incision laparoscopic-assisted appendectomy (SILAA) required more operative time than conventional methods, with an average operative duration of 65.3 minutes for SILAA compared to 56.5 minutes for CLA (p = 0.039)​ [3]. This suggests that while some minimally invasive techniques, like TULAA, may be advantageous in reducing operative time, others like SILAA may require more time due to technical challenges.

Postoperative pain was significantly lower in the case group of the present study (p = 0.0009), with 92% of patients reporting a pain score of 2 compared to 8.3% in controls. This aligns with findings from Boo et al. (2015), who reported that TULAA had significantly lower postoperative pain compared to SILA (p < 0.001)​ [12]. Similarly, Go et al. (2016) found that TULAA led to significantly lower postoperative pain scores and reduced the use of rescue analgesics compared to CLA​ [7]. Wu et al. (2022) also confirmed that SILA patients experienced less postoperative pain than those undergoing CLA​ [3].

The present study found that hospital stay was significantly less in the case group (p = 0.0008), with 76% of patients discharged within three days, compared to 24% in controls. This is consistent with Cheema et al. (2024), who reported that TULAA resulted in a shorter hospital stay than CLA (mean difference: -0.44 days, p = 0.002)​ [11]. Similarly, Cirocchi et al. (2024) concluded that single-port laparoscopic-assisted appendectomy (SILA) resulted in a shorter hospital stay compared to CLA, although the difference was not statistically significant​ [13]. However, Bindi et al. (2023) [9] and Noviello et al. (2015) [10] did not find a statistically significant difference in hospital stays between different laparoscopic techniques​. This suggests that while some studies demonstrate a benefit of newer techniques in reducing hospital stays, the evidence remains mixed.

Postoperative complications were noted in two cases in the present study, but the difference was not statistically significant (p = 0.1456). Previous studies have shown varied results regarding complication rates. Cheema et al. (2024) found that TULAA was associated with a lower risk of intraabdominal infections (relative risk (RR) = 0.64, p = 0.03)​ [11]. However, Wu et al. (2022) found no significant difference in wound infections between CLA and TULAA [3]. Boo et al. (2015) found that TULAA had a significantly lower complication rate (1.5%) compared to SILA (9.8%) (p = 0.0035)​ [12]. In contrast, Karam et al. (2016) reported a slightly higher surgical site infection rate for TULAA (6%) compared to CLA (4%), though the difference was not statistically significant​ [8].

Cosmetic acceptance was significantly better in the case group of the present study (p < 0.001). This aligns with findings from multiple studies, including Rometra et al. (2018) [14] and Cirocchi et al. (2024) [13], which emphasized that TULAA resulted in superior cosmetic outcomes compared to CLA​. Wu et al. (2022) also found that patients who underwent TULAA techniques had significantly higher cosmetic satisfaction scores than those who had CLA (p < 0.05)​ [3]. However, some concerns remain regarding the risk of umbilical deformity and incisional hernias in transumbilical SILA (TSILA), as highlighted by Wu et al. (2022)​ [3]. A small sample size limits the generalizability of findings, while the lack of long-term follow-up prevents assessment of sustained outcomes and late complications.

## Conclusions

The present study demonstrates that TULAA offers significant advantages over CLA in pediatric patients. TULAA resulted in a shorter operative time, reduced postoperative pain, faster recovery, and superior cosmetic outcomes, with no significant difference in complication rates. Given its minimally invasive nature and favorable surgical outcomes, TULAA should be considered a preferred approach for managing uncomplicated appendicitis, particularly in younger patients. However, further large-scale studies are required to validate its long-term efficacy and safety.
